# SOX2 haploinsufficiency promotes impaired vision at advanced age

**DOI:** 10.18632/oncotarget.26393

**Published:** 2018-11-30

**Authors:** Leire Moreno-Cugnon, Ander Anasagasti, Maitane Ezquerra-Inchausti, Ander Izeta, Pedro de la Villa, Javier Ruiz-Ederra, Ander Matheu

**Affiliations:** ^1^ Cellular Oncology Group, Biodonostia Health Research Institute, San Sebastián, Spain; ^2^ Sensorial Neurodegeneration Group, Biodonostia Health Research Institute, San Sebastián, Spain; ^3^ Tissue Engineering Laboratory, Biodonostia Health Research Institute, San Sebastián, Spain; ^4^ Visual Neurophysiology, IRYCIS, University of Alcala, Madrid, Spain; ^5^ RETICS OFTARED, Madrid, Spain; ^6^ IKERBASQUE, Basque Foundation for Science and CIBERfes, Bilbao, Spain

**Keywords:** Sox2, haploinsufficiency, aging, retina stem cell progenitors, vision loss

## Abstract

Age-related vision loss has been associated with degeneration of the retina and decline in Müller glia cell activity. Sox2 is a critical transcription factor for the development and maintenance of the mammalian retina. Here we determined the role of Sox2 in retinal aging. We observed a decline in the number of Sox2-positive Müller, amacrine and ganglion cells with age. We also explored the impact of *Sox2* haploinsufficiency (*Sox2^GFP^*) on the activity of Müller glia cells and vision loss with age. Reduction of Sox2-positive cells promoted impaired Müller glia cell function at advanced age of *Sox2^GFP^*. These findings correlated with a significant decline in electroretinographic response in *Sox2* haploinsufficient mice. Together, these results indicate that Sox2 is required for the maintenance of the transmission of visual information from cones and rods, and suggest that decline in Sox2 expression is responsible for retinal cell aging and age-related vision loss.

## INTRODUCTION

Visual deterioration occurs in healthy individuals with age, with impairment in acuity, contrast sensitivity, visual field and dark adaptation, among other changes, which have a dramatic effect on the quality of life in older people [1]. As individuals age, all mammalian ocular tissues undergo various anatomical changes. The internal membrane of the retina becomes thicker and neural elements with gliosis are decreased in the periphery of the retina [2]. Furthermore, aging induces a reduction in the nuclei in the outer nuclear layer of the retina. There is an age-related loss of rods in the macula, which leads to a decrease in scotopic sensitivity [2]. To function properly, the retinal overall size and the proportion of each retinal cell type must be strictly regulated. Among the seven retinal cell types, all derived from a pool of multipotent retinal progenitor cells (RPCs), Müller glia (MG) is the principal macroglia of the retina and maintain stem cell characteristics, including the expression of RPC markers [3]. With age, there is a decline in the number of retinal ganglion cells (RGCs) and interneurons, which comprise amacrine, bipolar and horizontal cells [4]. Moreover, deregulation of MG may be the primary mechanism for age-related retinal degeneration [5]. To date, the molecular mechanisms responsible for age-related vision loss remain largely unknown.

Sox2 (Sry-box containing gene 2) is a key factor in maintaining and inducing the pluripotency of embryonic cells. Moreover, it is highly expressed in stem cells both in embryonic and adult stages, and plays a critical role in the neural central system, including neural retina [6, 7]. In the retina, Sox2 is expressed in RPCs and its strict regulation is a critical factor for RPC differentiation during development [8]. Moreover, the expression of Sox2 is restricted to neural retinal cells [9] and it is required for the maintenance of quiescence at early postnatal MG [10–12]. Studies in mouse models of *Sox2* have shown that conditional ablation of this factor compromises the proliferation and differentiation capacities of RPCs, while hypomorphic levels cause an aberrant differentiation that leads to diverse microphthalmic phenotypes in postnatal animals [8]. The deficiency of *Sox2* in MG is associated with disruption of amacrine and horizontal cell neurites in the nuclear and outer plexiform layers, respectively, suggesting a role for Sox2 in the maintenance of retinal cytoarchitecture and function [3]. These results translate to human pathologies, with 10% of individuals with anophthalmia or severe microphthalmia having haploid insufficiency due to *SOX2* mutations [8].

Previous evidence supports the idea that Sox2 levels regulate RPC identity and differentiation in a dose-dependent way, but little is known about its effect on the retina with age. Thus, in this study, we explored the effects of *Sox2* haploinsufficiency in aged retina.

## RESULTS

### Aged *Sox2*-haploinsufficient mice have fewer numbers of certain retinal cell types

**Figure 1:**
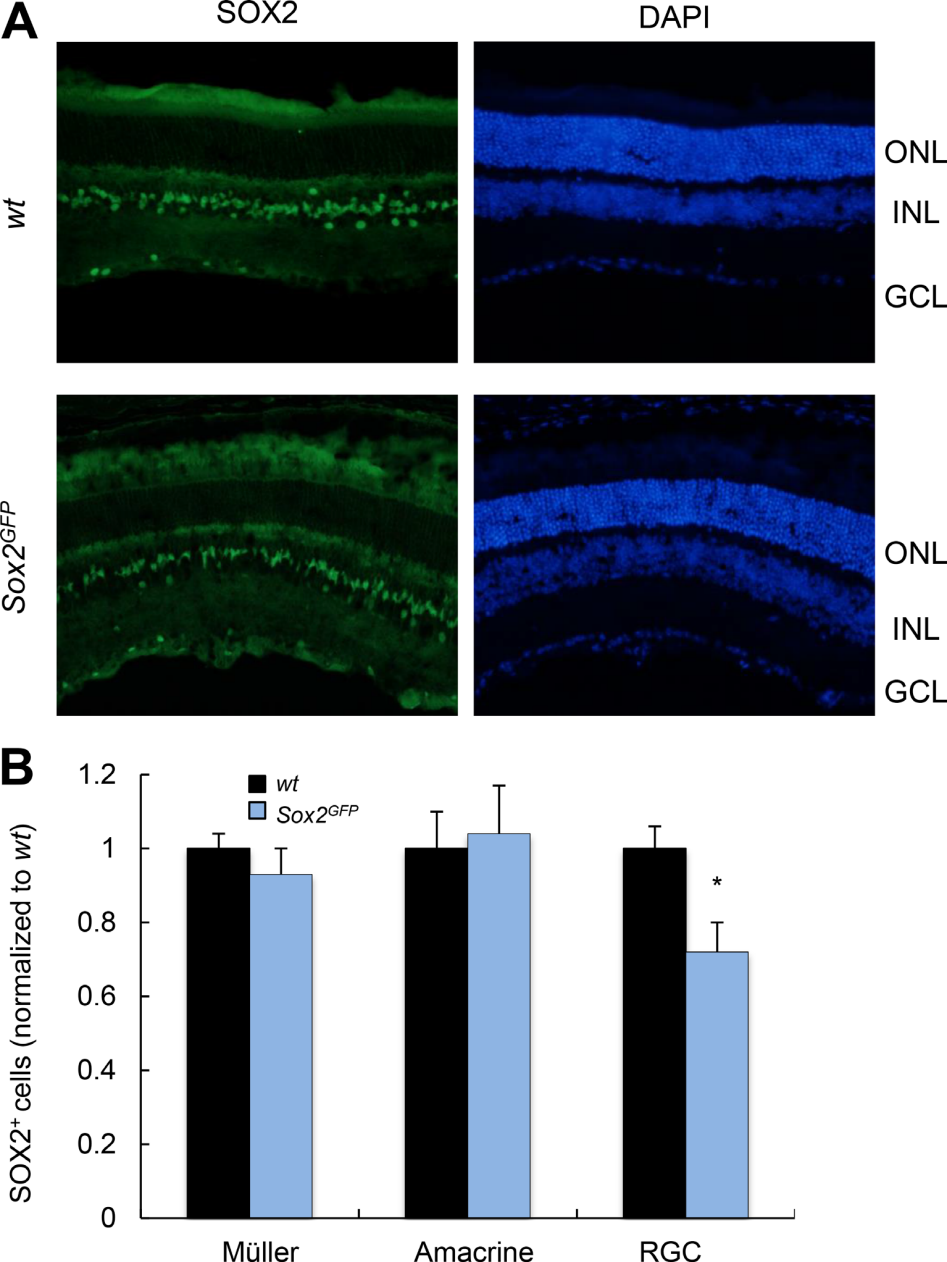
Young *Sox2*-haploinsufficient mice have normal numbers of retinal cells (**A**) Representative immunostaining of Sox2 in the retina of young *wt* and *Sox2^GFP^* mice. (**B**) Relative quantification of Sox2-positive staining in Müller, amacrine and ganglion cells in young (1–2 month-old) *Sox2^GFP^* relative to *wt* mice. ONL, outer nuclear layer; INL, inner nuclear layer; GCL, ganglion cell layer. Statistical differences (^*^p < 0.001) were assessed between genotypes by Student's *t* test. *n* = 3 mice, 6 retinas/group.

Given the role of MG in supporting retinal neurons, and that alterations in this cell type give rise to the disruption of axons and dendrites [3], we first determined the expression of Sox2 in MG, amacrine and ganglion cells (RGCs) in young *wt* and *Sox2^GFP^* mice. In young animals, *Sox2* haploinsufficient mice had similar numbers of Sox2-positive MG and amacrine cells compared to *wt* mice, but fewer RGCs (Figure 1A, 1B).

**Figure 2:**
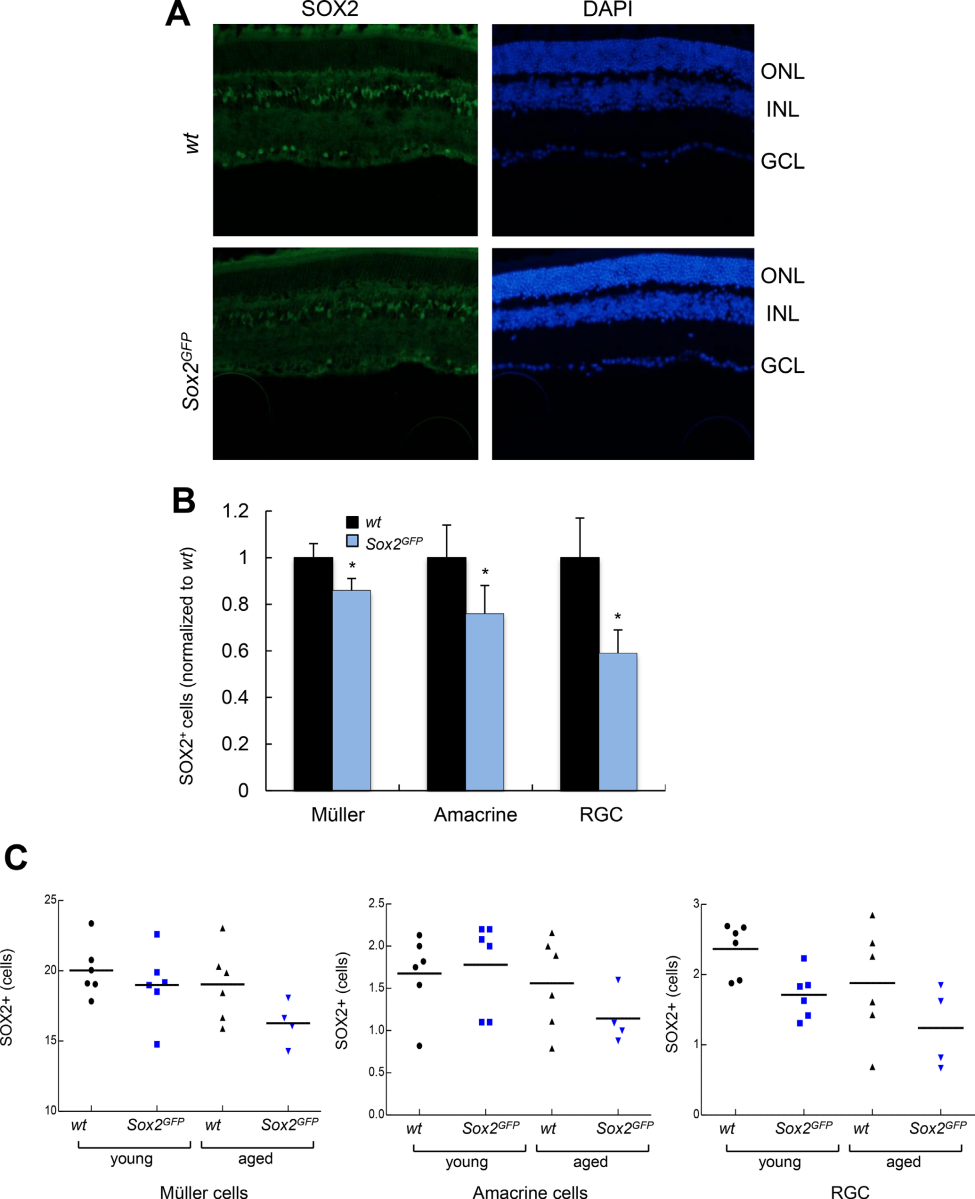
Aged *Sox2*-haploinsufficient mice have small numbers of certain retinal cell types (**A**) Representative immunostaining of Sox2 in the retina of aged (over 24 month-old) *wt* and *Sox2^GFP^* mice. (**B**) Relative quantification of Sox2 positive staining in Müller, amacrine and ganglion cells in aged (over 24-month-old) *Sox2^GFP^* compared to *wt* mice. (**C**) Quantification of the number of Sox2-positive cells in retinal cell types in *wt* and *Sox2^GFP^* mice at different ages. Statistical differences (^*^p < 0.05, ^**^p < 0.01) were assessed between genotypes by Student's *t* test. *n* = 3 mice, 6 retinas/group.

Next, we conducted the same analysis in over 21-month-old *Sox2^GFP^* and *wt* mice. Importantly, the relative numbers of MG, amacrine cells and RGCs positive for Sox2 were 0.86, 0.76 and 0.59 respectively, in aged *Sox2^GFP^* normalized to the numbers in aged *wt* mice (Figure 2A, 2B). When we compared mice of different ages, we detected smaller number of Sox2-positive cells among the different retinal cell types in aged mice of both phenotypes, *Sox2^GFP^* and *wt,* than in young mice (Figure 2C). However, the difference was more marked in Sox2-haploinsufficient mice, with the detection of fewer Sox2 positive cells corresponding to MG, amacrine cells and RGCs in aged *Sox2^GFP^* mice than in *wt* mice (Figure 2C). These results indicate that there is a significant decline in the number of Sox2-positive cells in different cell layers of the retina with age, which is aggravated in *Sox2* haploinsufficient mice.

### *Sox2* haploinsufficient mice have impaired visual function at advanced age

**Figure 3:**
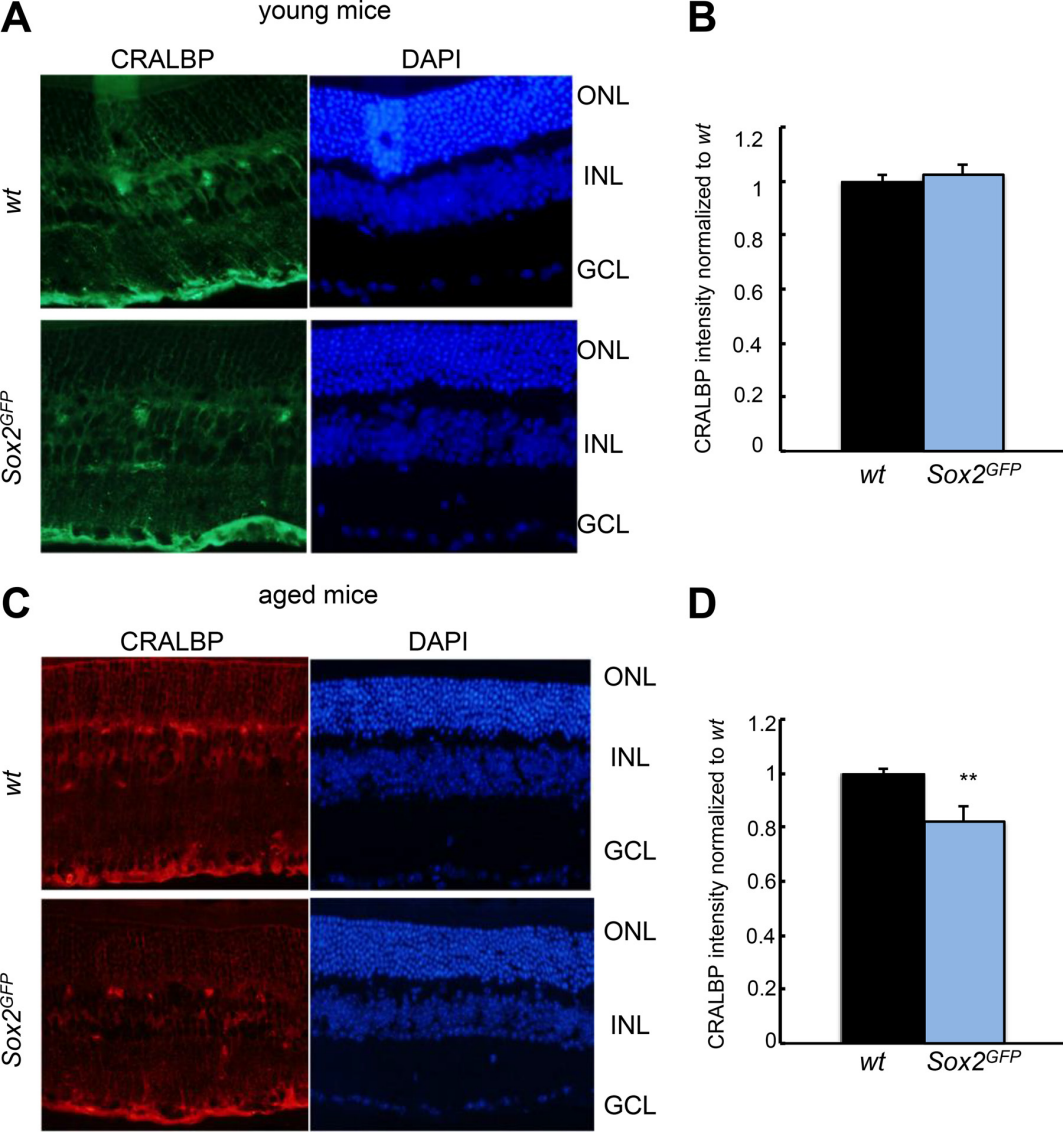
CRALBP expression is reduced in aged *Sox2*-haploinsufficient mice (**A, B**) Representative immunostaining (left) and relative intensity of CRALBP in young *Sox2^GFP^* compared to *wt* mice. (**C, D**) Representative immunostaining (left) and relative intensity of CRALBP in aged *Sox2^GFP^* compared to *wt* mice. Statistical differences (^**^p < 0.01) were assessed between genotypes by Student's *t* test. *n* = 3 mice, 6 retinas/group.

To assess changes in MG morphology and function, retinas from young (1–2 month-old) and aged (over 21-month-old) *Sox2^GFP^* and *wt* mice were stained with Cellular retinaldehyde-binding protein (CRALBP). The resulting images revealed that the structure of MG and intensity of staining was similar in young mice (Figure 3A, 3B). However, MG structure is less organized in *Sox2^GFP^* than *wt* mice. Moreover, there was less intensity in CRALBP staining in aged *Sox2^GFP^* than in *wt* mice (Figure 3C, 3D), features that might indicate an alteration in MG function in aged *Sox2* haploinsufficient mice.

**Figure 4:**
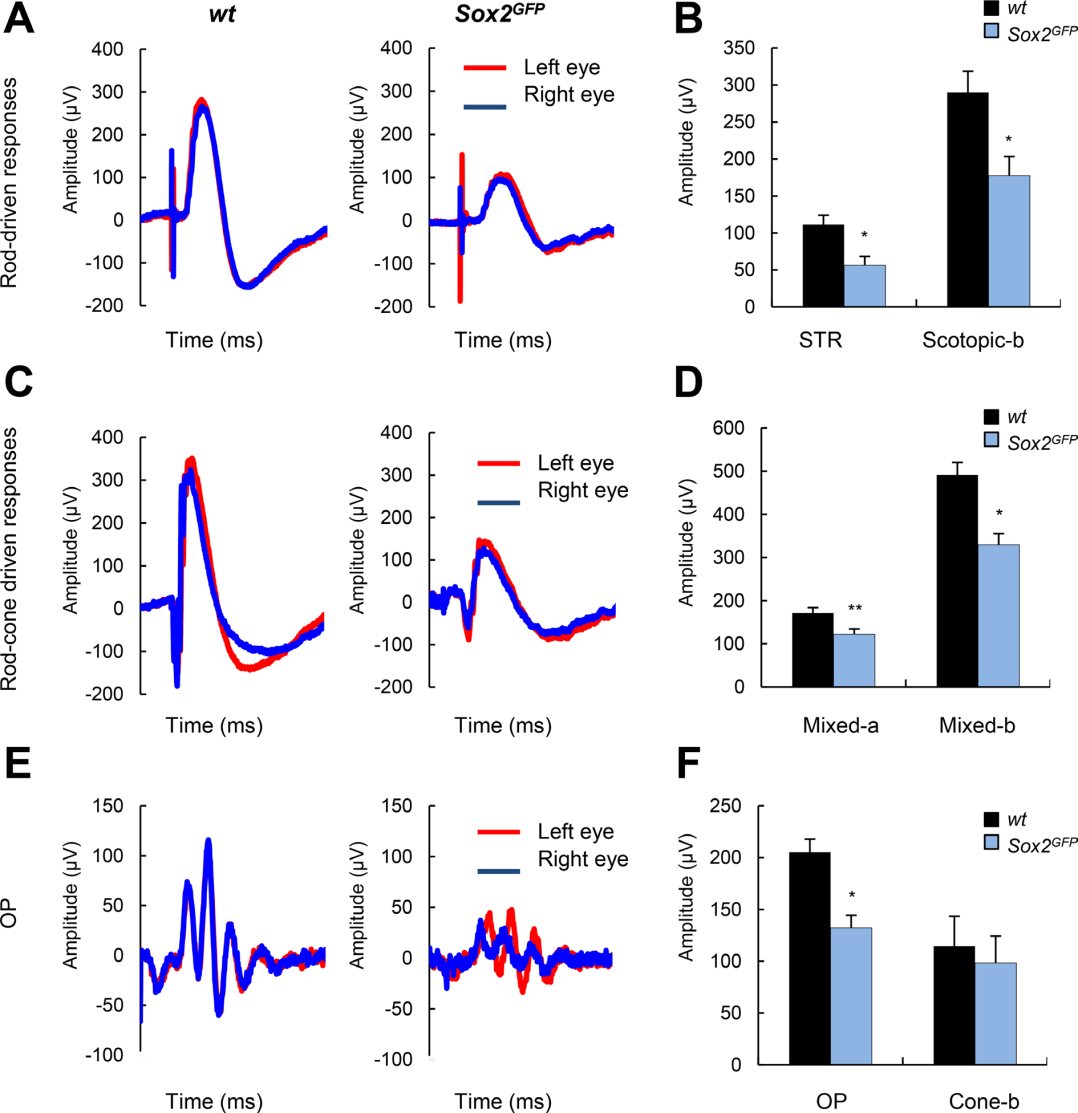
Aged Sox2-haploinsufficient mice have impaired visual function (**A**) Scotopic electroretinographic (ERG) (rod-driven) response elicited by a –2 log cd∙s∙m^–2^ flash stimulus in *wt* and *Sox2^GFP^* mice of around 1 year of age, recorded in left and right eyes (*n* = 3). (**B**) Histogram representation of scotopic threshold response (STR) and scotopic b-wave amplitudes. (**C**) Mixed ERG (rod plus cone) responses elicited by a 1.5 log cd∙s∙m^–2^ stimulus under scotopic conditions. (**D**) Cone responses elicited by a 1.5 log cd∙s∙m^–2^ stimulus under photopic conditions. (**E**) Histogram representation of mixed a- and b-wave amplitudes (**F**) Histogram representation of OP and cone b-wave responses under photopic conditions. Statistical differences (^*^p < 0.05, ^**^p < 0.01) were assessed between genotypes by Student's *t* test.

To determine the effect of *Sox2* haploinsufficiency on visual function, we evaluated retinal physiology in *wt* and *Sox2^GFP^* mice by recording electroretinographic (ERG) responses. Figure 4 shows the scotopic (i.e., rod photoreceptors activity in dark-adapted mice) and photopic (i.e., cone photoreceptors activity in light-adapted mice) ERG responses induced by different light intensities in mice of both *Sox2^GFP^* and *wt* genotypes at advanced age. *Sox2^GFP^* mice showed weaker ERG responses than *wt* mice to light intensities of –2 log cd·s/m^2^ and 1.5 log cd·s/m^2^ recorded in dark-adapted conditions. Mean data on rod-driven scotopic threshold response (STR) showed significant differences between genotypes, with response amplitudes of 111.29 μV in *wt* and 56.28 μV in *Sox2^GFP^* mice (Figure 4A, 4B). Similarly, when the mixed responses of cones and rods were compared (indicated on the graph as scotopic-b, mixed-a and mixed-b), the amplitudes of response were smaller in haploinsufficient than *wt* mice (Figure 4C, 4D). We also assessed the ERG responses of aged *wt* and *Sox2^GFP^* mice under photopic conditions. The ERG b-wave (a reflection of ON bipolar and Müller cell activity) was found to be smaller in *Sox2^GFP^* than in *wt* mice (Figure 4E). Moreover, we detected smaller amplitude in the oscillatory potential (OP), which refelcts amacrine cell activity; and in the response of rods and rod-associated cells in *Sox2^GFP^* mice (132.30 μV vs 205.19 μV in *wt* mice; Figure 4F). Taken together, our results show aberrant signal transmission from cone photoreceptors to cone bipolar cells in mice with *Sox2* haploinsufficiency, a condition that is associated with aging. Moreover, *Sox2^GFP^* mice dispalyed an impaired vision at advanced age.

## DISCUSSION

In this study, we show the essential role for Sox2 in the age-associated decline in retinal cell function and visual activity. During vertebrate eye development, the expression of Sox2 must be strictly regulated at all major stages of retinal development [10]. Moreover, this transcription factor strictly regulates the choice between maintenance of retinal progenitor cell identity and differentiation [8]. In the adult retina, MG constitute the principal cell type for the maintenance of retinal homeostasis [13] and their loss has been reported to be associated with retinal degeneration with age [14]. In this work, we revealed a decline in Sox2 expression in MG with age and showed this decrease impairs the activity of these cells. These results indicate that SOX2 might play a critical role in the maintenance of retinal stem cells with aging. In line with a putative role of Sox2 in stem cell aging, it has been recently reported that Sox2 expression decreases with age in neurogenic human and mouse brain samples [18], where Sox2 is a well-established stem cell maker of neural stem cells, and it plays a critical role in the maintenance of their identity [7, 15–17]. Moreover genetically modified mice that show delayed neural stem cell exhaustion, impaired neurogenesis and increased lifespan, have higher levels of Sox2 expression in the neurogenic niches [19].

Our results uncover a novel role for Sox2 in driving the age-associated deregulation and depletion of MG, extending the critical role of Sox2 in regulating MG at postnatal stage. Thus, *Sox2* ablation in MG resulted in postnatally retinal disorganization and degeneration [20–23]. Further, we have observed an age-associated decline in Sox2 expression in amacrine and ganglion cells. These results provide additional evidence of the impact of Sox2 in aged-associated retinal cell dysfunction. As in the case of MG, Sox2 is expressed in amacrine cells, where its activity is required for postnatal immature amacrine cell function and *Sox2* deletion leads to aberrant development [3, 24]. We have also detected a reduction in the number of Sox2 ganglion cells in *Sox2^GFP^* mice retina, consistent with previous studies where low levels of Sox2 have been associated with the loss of retinal ganglion cells [8]. In line with this, astrocytic loss of Sox2 affects the vascular architecture in maturity [25]. Together, our results demonstrate the essential role of this factor for the maintenance of cellular homeostasis in the retina with age.

Our data show a reduction in ERG b-wave amplitudes under scotopic and photopic conditions in aged *Sox2^GFP^* mice, suggesting impaired signal transmission from cones to bipolar cells. We also found that aged *Sox2^GFP^* mice have impaired visual function, indicating the critical role that Sox2 plays in age-related vision maintenance. Prior to our work, ablation of *Sox2* during postnatal retinal maturation in MG had been shown to give rise to structural abnormalities of the retina associated with a decrease in b-wave amplitude, which has been linked to its origin in MG [21]. Moreover, *Sox2* ablation postnatally was found to result in a significant decrease in ERG a- and b-wave amplitudes at 1 month of age [3]. While these studies demonstrated the need for Sox2 for the maintenance of retinal function, our study determined the role of this transcription factor under the chronic effects of physiological aging. Our work highlights that precise regulation of Sox2 expression is critical for temporal and spatial regulation of several types of retinal cells, including retinal progenitor cells, and thereby retinal function. In line with this, there is a dose-dependent effect of Sox2 on retinal progenitor competence [8]. The relevance of these results is reinforced by the severe eye malformations present in patients with *SOX2* mutations [26, 27].

In summary, our work demonstrates the critical role for Sox2 in retinal function and age-related vision loss, activities that might provide an explanation for understanding how hypomorphic levels of Sox2 expression result in retinal defects in humans. Indeed, there is a hypothesis that proposes *Sox2* levels as predictor of disease in the retina [8]. This theory supports the idea that once the levels of SOX2 decreases up to phenotypic levels (threshold established in 40% of normal) disorders may start to develop [8]. Our results indicate that there might be a correlation between the severity of the phenotype and the levels of SOX2 expression, not only in disease but also during physiological aging.

## MATERIALS AND METHODS

### Mice

B6;*129S*-*Sox2^tm2Hoch^*/J mice, from now on *Sox2^GFP^* [28], were housed in a pathogen-free barrier facility at the Biodonostia Health Research Institute and handled in compliance with the regulations on animal research specified in the European Union Directive [2010/63/EU]. All experiments were approved by the Biodonostia Institute and University of Alcala animal care, research and experimentation ethic committees. Mice were used for the study without considering the gender of the animals.

### Tissue processing

Eyes from young (1–2 month-old) and aged (over 21-month old) mice were dissected and fixed in paraformaldehyde (PFA, 158127-5006, Sigma-Aldrich) for 24 h, and then stored in sucrose (Panreac) 10% for 2 h, 20% for 2 h and 30% for 1 week, before embedding them in OCT. A total of 6 retinas from 3 mice were analyzed per age and genotype. Serial sections of 7 μm were cut using a CM1950 Cryostat (Leica). An average of 15 sagittal sections/eye were obtained from central retina. Retinal sections were incubated with SOX2 (1:500, Neuromics) or CRALBP (1:500, abcam), overnight at 4°C. Secondary antibodies were incubated for 1 h at room temperature (1:1000, Thermo Fisher), together with 4′,6-diamidno-2-phenylindole (DAPI) to stain the nuclei. Retinal sections were then mounted with Fluoro-Gel (Electron Microscopy Sciences). Cell immunofluorescence was evaluated with an Eclipse 80i microscope and processed with NIS-Elements Advanced Research software (both from Nikon). Images from the entire retinal section were captured so that the total number of each retinal cells type was analyzed in each section. The total number of nuclei was determined using the DAPI channel. CRALBP intensity was evaluated using Image J Software.

### Electroretinography

ERG measurements were made in 10- to 12-month-old mice, by an observer blind to the experimental condition of the animals. Mice were dark-adapted (>12 h) and deeply anesthetized with an intraperitoneal injection of saline solution (NaCl 0.9%) containing ketamine (125 mg/kg; Imalgene; Merial, Pirbright, England) and xylazine (5 mg/kg; Xilagesic; Calier, Granollers, Barcelona, Spain). During the entire procedure, animals were handled under indirect dim red light (>620 nm) and their body temperature was kept at 37°C on a heating platform (Hot-Cold, Pelton Shepherd Industries, Stockton, CA). Before recording, the pupils of the mice were dilated by applying topically two drops of 1% tropicamide (Culircusi Tropicamida; Alcon Cusí S.A., El Masnou, Barcelona, Spain). ERG responses were recorded using four electrodes: left and right corneal electrodes (contact lens type) (Burian-Allen, Hansen Ophthalmic Development Lab, Coralville, IA) centered on the visual axis 0.5 mm from the cornea, a reference electrode placed in the mouth, and a ground electrode attached to the tail. To optimize electrical recording and to prevent corneal surface desiccation, a topical drop of 2% methyl-cellulose (Methocel, Ciba Vision, Hetlingen, Switzerland) was administered to each eye immediately before placing the corneal electrode.

Electroretinographic responses to light stimuli were recorded using a full-field electroretinography technique. Low-intensity (<−2 log Cd·s/m^2^) stimuli were applied in a Ganzfeld dome, which ensures homogeneous illumination of at least the central 120° of the retina, whereas for higher-intensity stimuli (>−2 log Cd·s/m^2^), a single light-emitting diode was placed close to each eye. The electrophysiological response recorded was amplified and filtered (CP511 AC amplifier; Grass Instruments, Quincy, MA), and then digitalized (ADInstruments Ltd, Oxfordshire, UK). The whole process was controlled with Scope version 3.8.1 software (Power Lab, ADInstruments Ltd). The dim scotopic response (DSR), and rod (scotopic-b), mixed (a- and b-wave), and oscillatory potential (OP) responses were recorded sequentially under dark background conditions, and cone (photopic-b) and flicker responses were recorded following 5 minutes of light adaptation with background white light (50 Cd/m^2^).

### Statistical analyses

All data were expressed as the mean ± standard error of the mean (SEM). Student's *t* test was used to assess differences between two groups in normally distributed data. All analyses were performed using GraphPad Prism 5 Software, version 5.01 (Graph Pad Software).
